# Early Symptoms and Treatment Outcomes in Neuronal Ceroid Lipofuscinosis Type 2: Croatian Experience

**DOI:** 10.3390/jpm14080783

**Published:** 2024-07-24

**Authors:** Jelena Radić Nišević, Ivana Kolić, Marija Kostanjski, Franka Kovačević, Igor Prpić

**Affiliations:** 1 Division of Child Neurology, Department of Pediatrics, Clinical Hospital Center, 51000 Rijeka, Croatia; franka.kovacevic@uniri.hr (F.K.); igor.prpic@uniri.hr (I.P.); 2 Faculty of Medicine, University of Rijeka, 51000 Rijeka, Croatia; ivana.kolic@uniri.hr (I.K.); mkostanjski@student.uniri.hr (M.K.)

**Keywords:** enzyme replacement therapy, epilepsy, neuronal ceroid lipofuscinoses

## Abstract

Background: Late infantile neuronal ceroid lipofuscinosis type 2 (CLN2) is a rare neurodegenerative disease that generally appears in children between 2 and 4 years old, leading to seizures and a progressive loss of language and motor functions. As the disease progresses, affected individuals typically experience blindness and ultimately pass away in late childhood. Treatment with intracerebroventricular cerliponase alfa has been shown to slow the deterioration of motor and language functions compared to the natural progression of the disease. We aim to highlight the early symptoms of CLN2 which help with early diagnosis and timely treatment initiation in children with specific medical indications, as well as identify medical contraindications for enzyme replacement therapy. Methods: We describe five Croatian patients and one Bosnia and Herzegovinian patient with CLN2 disease, analyzing the clinical characteristics, neuroimaging findings, electroencephalogram results, genetic analysis, treatment indications and contraindications, and disease progression. Results: All six patients presented with seizures: focal seizures (*n* = 1), myoclonic–atonic seizures (*n* = 1), febrile seizures (*n* = 2), and tonic–clonic seizures (*n* = 2), along with language delay (*n* = 6). Despite this, one patient refused treatment, two were initially included in the clinical trial and then continued treatment, one did not indicate starting treatment, and three continued treatment. One patient, after 4.5 years of treatment, no longer had medical indications for the therapy, which was discontinued. The other two patients who received treatment had a significant slowing of disease progression. Conclusions: The early onset of seizures between ages 2 and 4, alongside delayed language development, is a defining characteristic of CLN2 disease. Enzyme replacement therapy using cerliponase alfa represents the initial treatment for neuronal ceroid lipofuscinosis type 2, targeting the underlying cause of the disease. It effectively delays the progression of language and motor decline in patients diagnosed with this condition.

## 1. Introduction

The neuronal ceroid lipofuscinoses (NCLs) encompass a diverse group of lysosomal storage diseases, which are the primary cause of dementia in children [1]. Neuronal ceroid lipofuscinosis type 2 (CLN2), commonly referred to as Batten disease, arises due to mutations affecting both copies of the CLN2 gene. These mutations result in a deficiency of the tripeptidyl peptidase 1 (TPP1) enzyme [2,3], which under normal circumstances functions to cleave tripeptides from the N-terminus of peptides. This deficiency leads to the accumulation of storage material within lysosomes, culminating in neuronal loss. The most frequently observed mutations worldwide include c.622C>T [p.Arg208X] and c.509-1G>C [4], with an estimated incidence of less than 0.5 per 100,000 live births [5,6,7].

CLN2 typically manifests between ages 2 and 4 and is characterized by seizures, language delay, and ataxia [8]. Throughout the years of 2 to 3, rapid disease progression leads to pharmacoresistant epilepsy, movement disorders, a decline in developmental abilities, dementia, and visual regression [2,8,9]. By age 6, significant motor and cognitive decline occurs, including the onset of involuntary movement and changes in speech and swallowing [8]. Visual impairment due to retinal atrophy usually begins between ages 7 and 10, with median death occurring in mid-adolescence, typically around 10 years of age, according to the DEM-CHILD natural history registry [9]. Atypical variants of the classical phenotype may present with later onset and varying clinical features [10].

Determining the clinical characteristics of CLN2 disease involves identifying specific electroencephalogram (EEG) patterns triggered by intermittent photic stimulation (IPS) at slow flash rates (1–3 Hz) and observing magnetic resonance imaging (MRI) evidence of atrophy, confirmed by enzyme and genetic testing [11,12]. Despite its devastating impact, CLN2 diagnosis is often delayed because the disease is rare and clinicians have limited awareness, which impedes timely intervention. However, early diagnosis is crucial as it facilitates access to potential treatments, such as enzyme replacement therapy (ERT) with cerliponase alfa [13]. Since its regulatory approval in 2017 in Europe and the United States, cerliponase alfa has revolutionized the management of CLN2, significantly improving disease progression. In 2019, it received approval in Croatia and was reimbursed through health insurance [14]. Nevertheless, while ERT slows disease progression, it does not reverse the neurodegeneration already present, highlighting the importance of early diagnosis. Despite limited literature on the diverse presentation and treatment adjustments, our goal is to identify early CLN2 symptoms to facilitate prompt diagnosis and the initiation of treatment in children with specific medical indications, while also recognizing contraindications for enzyme replacement therapy.

## 2. Materials and Methods

This retrospective study examines a cohort of patients from Croatia diagnosed with CLN2 disease during the period from 2019 to 2024. The cases were collected from the center in Rijeka, Croatia, which serves as the national center for enzyme replacement therapy treatment. Informed consent was obtained from the legal guardians or parents of each patient. Data extracted from medical records included clinical assessments, neuroimaging findings, electrophysiological evaluations, and the results of metabolic and molecular investigations. Following initial symptoms, patients underwent a combination of enzymatic and genetic testing, confirming the diagnosis of CLN2 disease. Enzymatic testing involved a dried blood spot (DBS) assay to assess TPP1 enzyme deficiency, with testing for palmitoyl protein thioesterase 1 (PPT1) deficiency, the enzyme affected in CLN1 disease, used as a control. The high specificity of this test is noted by a leading CLN reference center [15]. Genetic confirmation utilized an epilepsy gene panel containing the TPP1 gene, facilitating quicker diagnosis.

## 3. Results

Six patients with CLN2 disease (five from Croatia, and one from Bosnia and Herzegovina) were included in the study: one boy and five girls. The diagnosis was confirmed using genetic (*n* = 6) and enzymatic (*n* = 6) methods. A classical phenotype was identified in all six patients. Two patients initially started treatment after diagnosis in clinical trials (Hamburg and Rome), and after ERT became available in Croatia, therapy was continued locally. One patient had no indication for starting treatment due to the loss of all developmental skills, epilepsy and dementia. Additionally, the parents of one patient declined treatment.

A table (Table 1) provides details on the age of onset of the first symptoms and their characteristics, time to diagnosis, age at diagnosis and symptoms prior to enzyme replacement therapy.

### 3.1. Patient Analyses

**Figure 1:**
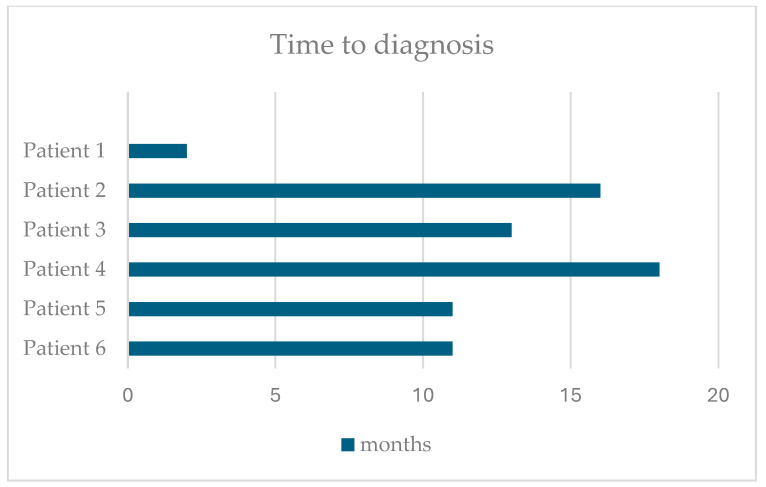
Time from the first symptom to diagnosis.

The median age at symptom onset was 3 years (range: 2 years, 6 months to 3 years, 8 months). Initial symptoms included focal seizures (*n* = 1), myoclonic–atonic seizures (*n* = 1), tonic–clonic seizures (*n* = 2), febrile seizures (*n* = 2), and language delay (*n* = 6). The median age at diagnosis was 3 years and 10 months (range: 3 years, 2 months to 5 years) with a median time from symptom onset to diagnosis of 11 months (range: 2 months to 18 months). One patient passed away at the age of 7 years and 10 months. Figure 1 illustrates the time from the first symptom to diagnosis for each patient.

### 3.2. Genetic and Enzymatic Diagnosis

A combination of genetic and enzymatic testing was conducted in all six patients. Recently, patients presenting with symptoms are initially diagnosed using gene panels, followed by confirmation through enzymatic testing. TPP1 enzyme activity testing revealed significantly decreased levels in all six patients: 0 nmol/spot*45 h in four patients (*n* = 4) and 0.01 nmol/spot*45 h in two patients (*n* = 2).

Genetic testing of the CLN2 gene was conducted for all patients. All patients (*n* = 6) were compound heterozygous; specifically, all patients exhibited the c.509-1G>C mutation. Three patients had this mutation alongside the c.622C>T (p.Arg208*), two had it alongside the c.622C>T (p.Arg208Ter) mutation, and one patient had it alongside the c.614T>A mutation. These results align with earlier research, such as the findings from the DEM-CHILD registry [10]. Table 2 presents the data for CLN2/*TPP1* gene sequencing.

### 3.3. Electroencephalogram

Comparing the EEG findings of the patients, in Patient 1, there are generalized irregular spikes during intermittent photic stimulation (IPS), while Patient 2 exhibits sporadic spike wave complexes predominantly frontal with propagation posteriorly. Patient 3′s recordings reveal sporadic generalized spike–wave bursts, particularly in deep sleep stages, whereas Patient 4 displays continuous spike–wave bursts, more pronounced on the left, and occasional high-voltage sharp waves during sleep. Patients 5 and 6 present generalized bursts of polyspike–wave complexes during sleep, along with occasional lower-voltage temporal spikes.

### 3.4. Brain MRI

Brain MRI was performed for all six patients (*n* = 6). Among the six patients who underwent MRI, findings varied. The median time from the onset of the first symptoms to the first MRI was one month. Two patients (*n* = 2) exhibited malformations in the posterior fossa; Patient 1′s MRI revealed a voluminous display of the intracranial segment of the clivus, which impinged on the area of the sphenoid sinus. Patient 2 had an atypical rotation of the hypoplastic vermis in the inferior segment, an enlarged fourth ventricle communicating widely with the enlarged cisterna magna, mild thinning of the corpus callosum, and disruption of white matter myelination, particularly in high frontoparietal and bilateral occipital regions. Patient 3 showed asymmetry in the lateral ventricles, and Patient 4 had a slightly different MRI profile, with normoposition of the ventricular system and a slightly enlarged fourth ventricle, distinct from the malformations observed in Patients 2 and 3. The MRIs of Patients 5 and 6 indicated hypoplasia of the vermis.

### 3.5. Enzyme Replacement Therapy (ERT)

In Croatia, after the proposed criteria from the national committee were accepted, treatment with cerliponase alfa was introduced by the Croatian Health Insurance Fund in 2019.

The criteria for initiating cerliponase alfa treatment include confirming CLN2 diagnosis through clinical, genetic, and enzymatic activity testing, achieving a CLN2 Rating Scale Motor-Language Score of 2 or higher, and excluding other progressive life-limiting conditions. Treatment initiation requires meeting all initial criteria, and patients must commit to attending medical appointments and complying with monitoring requirements.

Patients are excluded from cerliponase alfa treatment if they have clinically significant concurrent diseases or conditions that would not benefit from CLN2 treatment, or if they exhibit end-stage manifestations of CLN2 where therapy is unlikely to be beneficial. The first reassessment for patients receiving ERT with cerliponase alfa occurs after 18 months of treatment, followed by subsequent evaluations every 12 months.

The criteria for discontinuing cerliponase alfa treatment include a decline of three or more points on the CLN2 Rating Scale Motor-Language Score compared to baseline within the initial 18 months of treatment. Additionally, treatment cessation is warranted if the total CLN2 rating scale score falls below 2 after at least three infusions and persists for at least 6 weeks. Reductions in proxy-reported patient quality of life within the 18 months of treatment, as indicated by specific score declines, also justify treatment discontinuation. These stopping criteria specifically apply to patients starting treatment at age 3 or older, with children under 3 excluded until they reach this age and undergo baseline assessment. The treatment is halted if there is a progression of the disease, resulting in an irreversible assessment of 0 according to the CLN2 Rating Scale Motor-Language Score.

Patients scoring 0 should undergo reassessment twice within 12 weeks to verify that the decline is not due to temporary illness [https://hzzo.hr/zdravstvena-zastita/lijekovi/objavljene-liste-lijekova (accessed on 23 May 2024)].

The Hamburg scale, developed specifically for CLN2 disease, aims to measure the decline in function associated with disease progression. This scale consists of four components: motor function (walking ability), language, visual function, and seizures, each rated on a scale from 0 to 3. The total score ranges from 0 to 12. The CLN2 Rating Scale ML Score, which incorporates visual function and seizure items from the Hamburg scale along with modified versions of motor function and language items, has been adapted to maintain standardized evaluation across multinational and multisite clinical efficacy trials. Comparative analyses between the CLN2 Rating Scale ML Score and the Hamburg scale’s ML ratings have shown sufficient similarity, as detailed in Table 3 [16].

In our study, four out of six patients (*n* = 4) underwent enzyme replacement therapy. These patients include Patient 1, Patient 4, Patient 5, and Patient 6, as listed in Table 1. The median age at the onset of the first symptoms among treated patients was 2 years and 10 months, while the median age at diagnosis was 3 years and 6 months. Epilepsy was the initial symptom in all four cases within this subgroup of patients, with motor regression being an additional initial symptom in two cases, alongside epilepsy.

**Figure 2:**
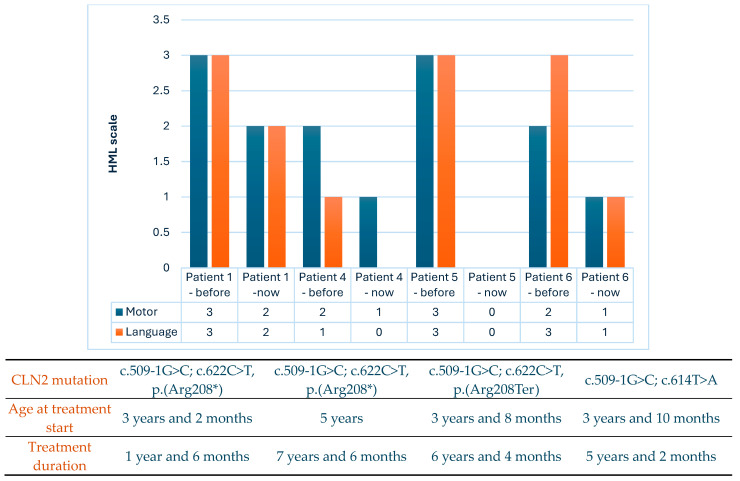
The HML scale score values before enzyme replacement treatment and at the cutoff date (18 May 2024) for four patients treated with cerliponase alfa. The table below describes mutation, age at treatment start, and duration of therapy. Abbreviation: HML, Hamburg motor and language.

Figure 2 illustrates the Hamburg scale values before enzyme replacement treatment and after for treated patients. Boxes describe CLN2 mutation, age at treatment start, and treatment duration.

**Figure 3:**
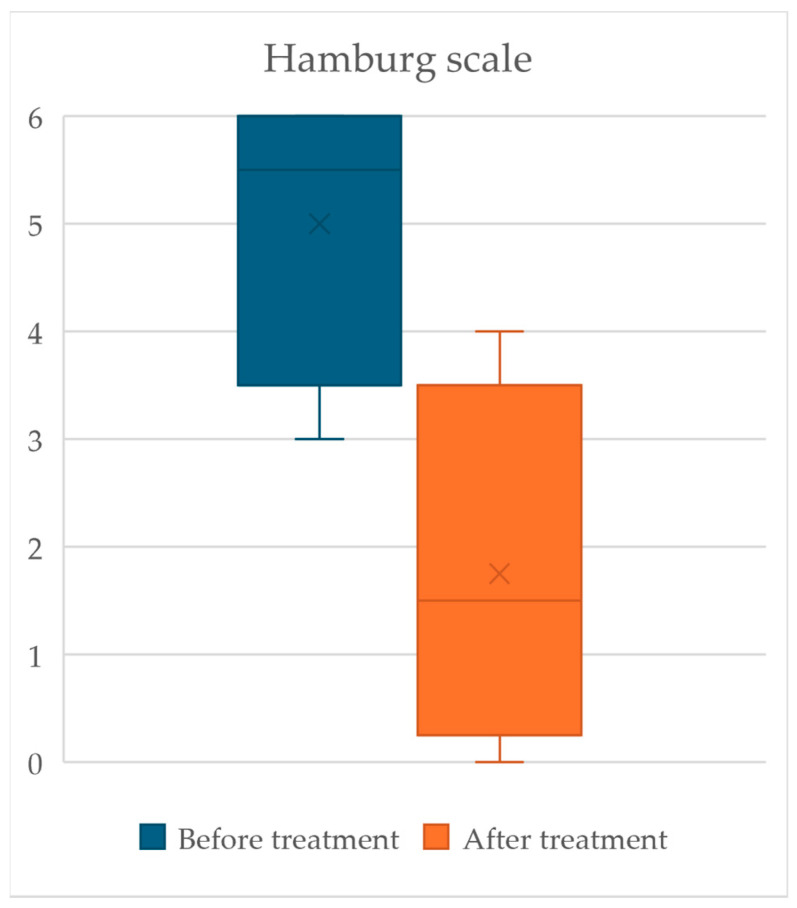
Box and whisker plots illustrating Hamburg scale scores before and after cerliponase alfa treatment depict median values (center lines), with the 25th and 75th percentiles represented by the box boundaries. The whiskers extend to values within two-thirds of the interquartile range (IQR).

The median Hamburg scale score before cerliponase alfa treatment was 5.5 points (range: 3 to 6 points). After treatment, it decreased to 1.5 points (range: 0 to 4 points), as illustrated in Figure 3.

The scale at the beginning of therapy for Patient 1, at the age of 3 years and 2 months, was 6 (motor 3, language 3), and it decreased to 4 (motor 2, language 2) after 1 year and 6 months. The child currently experiences progressing motor dysfunction, characterized by tremors that significantly affect the precision of movement direction. Nonetheless, the child manages to perform manipulative tasks. There is mild developmental delay overall, with an uneven developmental profile spanning from 2 to 4 years of age.

For Patient 4, starting therapy at the age of 5 years resulted in a scale of 3 (motor 2, language 1), which decreased to 1 (motor 1, language 0) after 7 years and 6 months of therapy. While the condition remains stable, there has been significant regression in speech and motor skills. The child does not walk and primarily communicates through nonverbal gestures. Additionally, they have become blind.

The scale for Patient 5 at the beginning of therapy at the age of 3 years and 8 months was 6 (motor 3, language 3), which decreased to 0 (motor 0, language 0) after 6 years and 4 months. The condition remains stable, but therapy is no longer administered due to a lack of indications—the patient no longer speaks and cannot walk independently. According to the stopping criteria for cerliponase alfa treatment, therapy is discontinued due to a decline of more than 1 point compared to the assessment in the previous 12 months.

The scale for Patient 6 at the beginning of therapy at the age of 3 years and 10 months was 5 (motor 2, language 3), which decreased to 2 (motor 1, language 1) after 5 years and 2 months. The child is currently incapable of vocalization/verbalization. She walks unsteadily with an intention tremor, and her vision is impaired.

Clinical deterioration, according to the scale, occurred in treated patients at the age of 5 years and 9 months (range: 1 year and 6 months to 7 years and 6 months). In untreated patients, clinical deterioration was observed at the age of 1 year and 5 months. During follow-up, no clinically significant complications related to therapy were reported. All patients showed positive outcomes, including stabilization or significant slowing of the disease course compared to its natural progression. No difference was on electroencephalography and brain MRI before and after treatment.

## 4. Discussion

We report early symptoms and evolution, treatment initiation according to inclusion criteria, and contraindications for treatment in children with CLN2.

Our cohort exhibited a median age of initial clinical symptoms comparable to the DEM-CHILD and Weill Cornell Medical College datasets (36 months vs. 35 months) and a shorter time from symptom onset to diagnosis (11 months vs. 22.7 months). The median age at diagnosis in our cohort was 46 months, compared to 54 months in the referenced datasets [12]. These differences are likely affected by increased awareness and improved access to enzymatic and genetic testing in clinical practice. Recent studies underscore the benefits of early epilepsy gene panel testing, reducing diagnostic delays (9.8 months vs. 22.7 months) and enabling timelier intervention [14].

Seizures and delayed language development were predominant initial symptoms in our cohort (present in all six patients), aligning with the existing literature.

Understanding specific language deficits in CLN2 disease, such as limitations in sentence progression, delayed vocabulary expansion, and halted language development [16], could aid early detection and care. The recognition of language delay, especially concurrent with initial seizures, warrants suspicion for CLN2, prompting enzymatic testing.

Early photosensitivity (a typically photoparoxysmal response at the low stimulation frequencies of 1–3 Hz) is a hallmark of CLN2 disease [13]. In five of our patients, IPS at low frequencies was performed at the initial diagnostic work-up, and the results were positive. In one patient, it was not performed appropriately due to non-cooperation. Photosensitivity, as an early CLN2 disease feature, should be a standard procedure in every EEG laboratory, and especially performed in young children presenting with any type of seizure and delayed language development.

Although there is sparse literature concerning subtle MRI findings in the early stages of CLN2 disease, studies have identified progressive cerebellar and cerebral atrophy, as well as changes in the periventricular white matter, as notable indications of this condition. Key MRI findings could be present at first seizure, but the signs can be subtle and go unrecognized, and be interpreted as “physiological” [17,18].

Historically, treatment for CLN2 has involved a multidisciplinary approach aimed at providing symptomatic relief and palliative care [19]. However, since the FDA and EMA approval of cerliponase alfa in 2017 as the sole medication for CLN2 treatment, efficacy in altering the disease’s natural progression has been documented [15,19]. In the natural course of the disease, the average estimated annual decline in clinical scores was 1.81 units. Once significant functional loss occurred, the decline accelerated from a score of 5 to 1, at a rate of 2.43 units per year, indicating nearly linear progression. These findings underscore the crucial significance of timely diagnosis and immediate initiation of treatment, given that the disease exhibits its most rapid progression shortly after the onset of initial symptoms [10].

In our center, we established specific inclusion and exclusion criteria to ensure that patients receive appropriate and effective treatment. These criteria help in identifying suitable candidates for therapy, optimizing treatment outcomes, and minimizing potential risks. An open-label, multicenter study evaluated the effects of biweekly intracerebroventricular infusions of cerliponase alfa on motor–language function. The findings indicated a significantly slower decline for treated patients (0.38 ± 0.10 points) compared to the natural history cohort (2.06 ± 0.15 points) [20,21,22]. In our cohort of four patients receiving treatment with cerliponase alfa, the median period for a 2-point decline in the CLN2 Rating Scale Motor-Language Score was 5 years and 9 months (range: 1 year and 6 months to 7 years and 6 months). In two untreated patients, clinical deterioration (decline > 2 points at CLN2 Rating Scale Motor-Language Score) was observed after 17 months, and one of them passed away at the age of 7 years and 10 months, 2 years and 10 months after the diagnosis was established. In Patient 5, the treatment of cerliponase alfa was effective in slowing disease progression, but at the age of 10 years, after 6 years and 4 months of the treatment, it was discontinued according to the defined criteria for stopping treatment. Therapeutic interventions now focus on reducing the frequency and severity of neurological symptoms, delaying complications, and improving quality of life given the progressive nature of the disease.

Furthermore, CLN2 is a neurodegenerative lysosomal storage disorder that leads to blindness, with retinal degeneration continuing in patients receiving cerliponase alfa ERT [23]. A recent study indicated that despite intraventricular ERT, retinal degeneration in CLN2 patients progressed significantly, especially between the ages of 56 and 80 months. Therefore, retina-targeted therapies should be started early, ideally before or during the phase of rapid retinal decline [24]. The impact of early diagnosis of CLN2 is profound, offering the potential to significantly alter the disease trajectory, improve patient outcomes, and provide crucial support to affected families. Enhanced awareness, early screening, and timely intervention are key strategies in achieving these benefits.

## 5. Conclusions

The early recognition and diagnosis of this disease is crucial for initiating therapy as soon as possible and providing a better quality of life to affected children and their families. The ERT does not cure, but it does significantly slow down the disease progression, so clear criteria must be set for treatment discontinuation, as well.

## Figures and Tables

**Table 1:** Overview of patients’ clinical characteristics prior to starting enzyme replacement therapy.

Patient	Gender	First Symptoms	Age at First Symptoms (Years/Months)	Time to Diagnosis	Age at Diagnosis	Symptoms Prior Enzyme Replacement Therapy
1	F	Tonic–clonic epileptic seizures	3 years	2 months	3 years and 2 months	Language and motor regression
2	M	Tonic–clonic epileptic seizures, motor and language regression	3 years and 8 months	1 year and 4 months	5 years	Hypotonia, neurological regression, absence of reflexes, muscle spasms ^a^
3	F	Myoclonic–atonic seizures, irritability	3 years	1 year and 1 month	4 years and 1 month	Motor and speech regression to loss of verbal communication, hyperreflexia, tremor, hypotonia
4	F	Focal seizures with loss of consciousness, motor regression	3 years and 5 months	1 year and 6 months	4 years and 11 months	Developmental regression, motor, and language regression, vision loss
5	F	Febrile seizures followed by neurological deterioration	2 years and 6 months	11 months	3 years and 5 months	Motor and language regression
6	F	Febrile seizures, ataxia, motor regression	2 years and 8 months	11 months	3 years and 7 months	Progressive vision loss, tremor, language regression

^a^ Involuntary contractions of muscles, common in CLN2, distinct from epileptic seizures. They can disrupt rest and sleep, causing distress for children and families.

**Table 2:** *TPP1* gene sequencing and TPP1 activity.

Patient	Phenotype	Allele	TPP1 Activity (nmol/spot*45 h)	TPP1 Gene Mutation(s)
1	Classical	Compound heterozygous	0	c.509-1G>C; c.622C>T, p.(Arg208*)
2	Classical	Compound heterozygous	0	c.509-1G>C; c.622C>T, p.(Arg208Ter)
3	Classical	Compound heterozygous	0.01	c.509-1G>C; c.622C>T, p.(Arg208*)
4	Classical	Compound heterozygous	0.01	c.509-1G>C; c.622C>T, p.(Arg208*)
5	Classical	Compound heterozygous	0	c.509-1G>C; c.622C>T, p.(Arg208Ter)
6	Classical	Compound heterozygous	0	c.509-1G>C; c.614T>A

**Table 3:** Comparison between the Hamburg scale and CLN2 Rating Scale Motor-Language Score.

Category	Hamburg Scale *	Score	CLN2 Rating Scale ML Score
Motor	Walks normally ^a^	3	Grossly normal gait. No prominent ataxia, no patologic falls
	Frequent falls, obvious clumsiness	2	Abnormal gait, frequent falls, independent walk > 10 steps
	No unaided walking or crawling only	1	Requires external assistance to walk or can crawl only
	Immobile, mostly bedridden	0	
Language	Normal (individual maximum) ᵇ	3	Apparently normal language. Intelligible and grossly age-appropriate. No decline noted yet
	Has become recognizably abnormal	2	Loss of words, intelligible but abnormal language (worse than the individual maximum)
	Hardly understandable	1	
	Unintelligible or no language	0	
Visual	Recognizes desirable object, grabs at it	3	
	Grabbing for objects uncoordinated	2	
	Reacts to light	1	
	No reaction to visual stimuli	0	
Seizures	No seizure in 3 months	3	
	1–2 seizures in 3 months	2	
	1 seizure per month	1	
	>1 seizure per month	0	

Abbreviation: ML, motor and language. ^a^ Motor development in certain children has always been consistently abnormal. ᵇ In certain children, typical language development has never been observed. In these instances, the highest level of achievement in language was considered the baseline and rated as 3; any subsequent decline in language ability was then rated as 2. * The Hamburg scale, developed to assess the progression of CLN2 disease, evaluates four domains: motor function (ability to walk), visual function, speech, and seizures. Each domain is rated on a scale from 0 to 3, with a maximum score of 12. To maintain consistency in multinational studies, the Hamburg scale has been adapted to assess only motor and language functions, resulting in a score ranging from 0 to 6, aligned with the CLN2 Rating Scale ML Score.

## Data Availability

The data presented in this study are available on request from the corresponding author due to privacy.
